# Machine Learning-Based Process Control for Injection Molding of Recycled Polypropylene

**DOI:** 10.3390/polym17070940

**Published:** 2025-03-30

**Authors:** Joshua Krantz, Juliana Licata, Muntaqim Ahmed Raju, Peng Gao, Ruizhe Ma, Davide Masato

**Affiliations:** 1 Department of Plastics Engineering, University of Massachusetts Lowell, Lowell, MA 01854, USA; joshua_krantz@student.uml.edu (J.K.); juliana_licata@student.uml.edu (J.L.); gaop@wwu.edu (P.G.); 2 Department of Computer and Information Sciences, University of Massachusetts Lowell, Lowell, MA 01854, USA; muntaqimahmed_raju@uml.edu (M.A.R.); ruizhe_ma@uml.edu (R.M.); 3 Department of Engineering and Design, Western Washington University, Bellingham, WA 98225, USA

**Keywords:** injection molding, recycled polypropylene, machine learning, process control

## Abstract

The increased interest in artificial intelligence in manufacturing has driven the adoption of machine learning to optimize processes and improve efficiency. A key challenge in injection molding is the variability of recycled materials, which affects part quality and processing stability. This study presents a novel closed-loop process control approach for injection molding, leveraging machine learning to adaptively predict processing inputs and quality outcomes. The methodology was tested on five blends of recycled polypropylene (rPP), using artificial neural networks (ANNs), linear regression, and polynomial regression to model the relationships between material properties and process parameters. The dataset was split 80/20 into training and testing sets. The ANN model was implemented using TensorFlow and Keras, with six hidden layers of 32 neurons per layer, ReLU activation, and an Adam optimizer. Empirical tuning and early stopping were used to optimize performance and prevent overfitting. Predictions were evaluated based on mean absolute error (MAE), mean squared error (MSE), and percentage error. The results showed that yield stress, ultimate elongation, and part weight were accurately predicted within a 5% error for linear and polynomial regression models and within a 10% error for the ANN. However, modulus predictions were less reliable, with errors of ~11% for ANN and linear regression and ~40% for polynomial regression, reflecting the inherent variability of this property in rPP blends. Predictions of processing inputs had errors ranging from 3% to 25%, depending on the model and response variable. No single modeling approach was consistently superior across all responses, highlighting the complexity of the relationship between material properties, process parameters, and quality metrics. Overall, the work demonstrates that closed-loop process control, powered by machine learning, can effectively predict key quality parameters in injection molding of recycled materials. The proposed approach can improve process stability and material utilization, facilitating increased adoption of sustainable materials.

## 1. Introduction

The rise of Industry 4.0 has led to increased technological advancements throughout most manufacturing industries. Injection molding is the largest plastics manufacturing industry in the world, with a total market size of 261.8 billion in 2021 [1], and it has been heavily influenced by this technological uprising [1]. This has increased production efficiency by reducing scrap rates, reducing material consumption, and minimizing cycle times. In this context, the use of machine and mold sensors [2], as well as machine learning, offer opportunities for advanced process control strategies [3,4].

Ensuring product quality while minimizing the cost of injection molded parts has been an area of focus in the industry since its inception. However, current practices involve the setting of processing variables by process engineers. The influence of these process settings on overall product quality has been researched extensively, and therefore, the overall product quality is heavily influenced by the expertise of the process engineer [5,6]. Hence, current research has focused on reducing reliance on processing expertise [7]. A tool often used for process optimization is multivariate regression analysis, as regression is an effective statistical method for determining a relationship between multiple dependent and independent variables [8]. Multivariate regression models are often used in injection molding to predict the significance of processing factors on part quality metrics (dimensional stability, weight) [9,10] and part performance metrics (mechanical properties) [11,12]. Regression modeling involves selecting one or more independent variables that will influence the response of one dependent variable. Statistical analysis is then performed to estimate the coefficients of each independent variable, resulting in the lowest error. The fit of the regression model is then validated, and the model fit is evaluated most commonly by the coefficient of determination (R^2^) value. The results of the model are an equation that takes the following form [13]:

(1) $$Y=\beta_{0}+\beta_{1}X_{1}+\beta_{2}X_{2}+\ldots +\beta_{n}X_{n}+\varepsilon ,$$

where *β*_0_ is the intercept, *β*_1_, *β*_3_, and *β_n_* are the coefficients for each independent variable (*X*_1_, *X*_2_, *X*_3_, *X_n_*), Y is the dependent variable, and ε is the error. The ease of application and ease of interpretation are key advantages of this method, as the coefficients attached to each predictor show how each predictor influences the final predicted response [13]. The main drawback of using linear regression modeling is that it attempts to find linear relationships between dependent and independent variables, whereas, in injection molding, the relationships are often complex and not linear [14,15]. To combat this drawback, additional regression models were evaluated, including a polynomial regression model, as follows [13]:

(2) $$Y=\beta_{0}+\beta_{1}X+{\beta_{2}X}^{2}+\ldots +{\beta_{n}X}^{n}+\varepsilon ,$$

where the variables are the same as those in linear regression, except that the independent variable is raised to the nth degree. The polynomial regression extends linear regression by introducing polynomial terms of independent variables, making the model nonlinear in terms of relationships but still linear in its parameters.

The quick rise of artificial intelligence in all aspects of industry has led to increased focus and use of these technologies as an opportunity for increased efficiency and process improvement [7]. As a result of this rise, the use of different machine learning algorithms within the plastics industry has drawn significant interest. Additionally, it has been combined with other technologies, such as simulations, to predict the quality of injection molded parts [7]. Research has been conducted on training ANN to predict resulting weight and warpage from different combinations of processing settings and part geometry [16,17]. Other research involving ANN has shown its efficacy in predicting various materials’ mechanical properties [18,19]. Moreover, research has been performed to determine the optimal prediction model by comparing multivariate regression modeling and ANNs [20]. The results showed that ANNs effectively captured nonlinear relationships, though performance varied across different quality metrics and in some cases, regression models performed comparably or better [21]. Table 1 highlights recent research papers. It can be observed that most research has focused on virgin resins, as well as using part weight and dimensional stability as the main quality parameters. Different machine learning algorithms, such as ANN, multivariate regression, and transfer learning, have been used to improve processing consistency, minimize startup time, maximize efficiency, and improve product quality.

Research conducted on ANNs has proved to be an effective way of reducing the amount of trial and error when establishing a process and reducing the reliance on human expertise. Indeed, ANNs do not require explicit programming for their creation and can take data and use training and learning to create a model, thus reducing the setup time and increasing efficiency [22]. ANNs work well with plastic injection molding due to the neural network’s capability to model non-linear behavior, encompassing the process and its relationship with different processing parameters. A basic ANN uses computing cells (i.e., neurons) that receive inputs; each of these is multiplied by a given weight, which determines the importance of that specific input in relation to the neuron. Additionally, an adder is used to sum all the input signals with their respective weights, as well as a bias to produce an output. An activation function is used to introduce nonlinearity to limit the range of the output signal to a finite value [23]. An ANN can predict parameters from a set of inputs through training. During training, backpropagation is used to compute the gradient of the error with respect to the network’s weights, which are then adjusted by an optimization algorithm to minimize the error. This process continues iteratively until a stopping criterion is met, improving the ANN’s ability to generalize to hold-out data [24]. Proper training and setting up of the ANN are crucial for accuracy. Overfitting (memorizing noise rather than learning patterns) and underfitting (failing to capture relevant patterns) can lead to inaccuracies. Additionally, hyper-parameter tuning is important as using the correct activation function, number of hidden layers, etc., is crucial to achieving accurate predictions and preventing overfitting [25].

Alongside the technological growth in the injection molding sector, there is a push for sustainable business practices, with particular emphasis on reduced energy consumption and the push for recycled materials [26,27]. The rise in recycled material usage has led to an increased reliance on appropriate process control methodologies to combat the inherent variability of these materials. This variation arises due to a permanent breakdown of the molecular structure, which decreases molecular weight and leads to a broader molecular weight distribution [15]. Machine learning models can be used to tackle the compositional variation in recycled materials at the process control level to maximize consistency [28]. Currently, most process control strategies focus on regression analysis; hence, the introduction of machine learning models represents an opportunity for further development [29].

In this work, a process control methodology was developed for a process that automatically adjusts in response to recycled material processing variability. Different data modeling approaches are evaluated for the closed-loop process control in injection molding. The methodology involves comparing the predictive performance of an ANN, linear regression analysis, and polynomial regression analysis. The models predict key injection molding processing inputs based on different quality response variables, including tensile properties and part weight. The work focuses on recycled polypropylene (rPP), which is one of the most commonly recycled and available thermoplastic resins.

## 2. Materials and Methods

### 2.1. Process Control Approach

**Figure 1:**
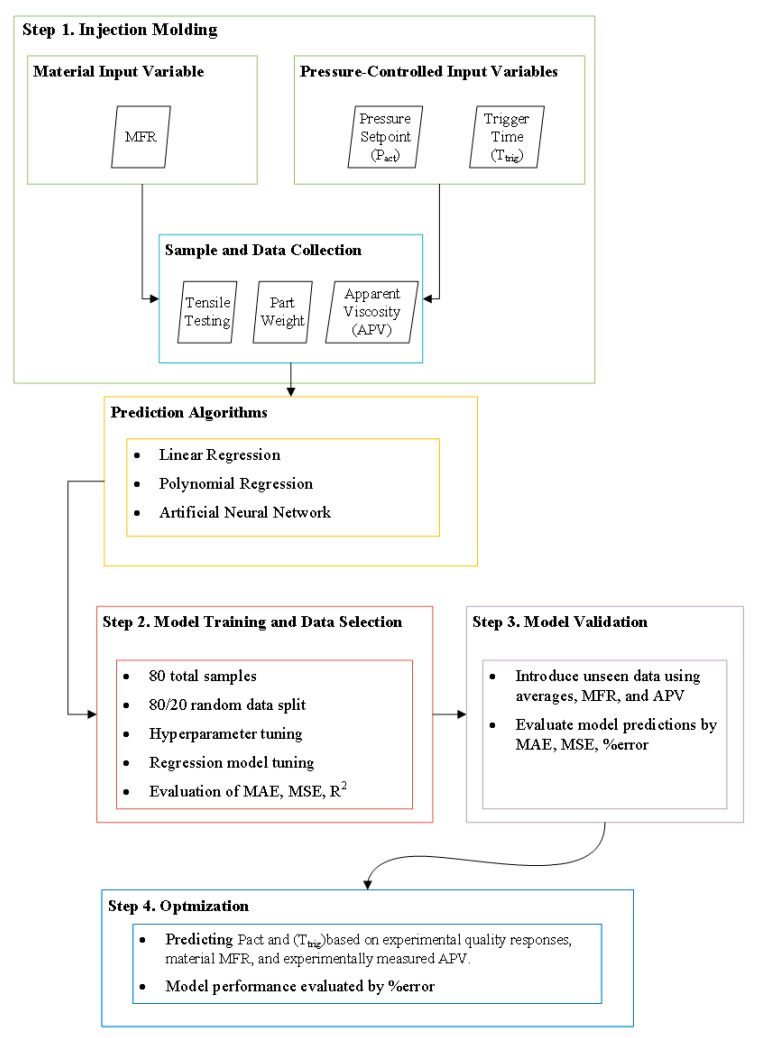
Flow chart depicting the methodology followed in this work.

Most research on machine learning in injection molding focuses on training and validating models to predict quality metrics (most frequently weight and dimensions) from combinations of process parameters (e.g., temperature, injection velocity, packing, etc.). This work focuses on the correlation between process inputs, in-mold sensing data, and quality metrics. The goal is to develop process control strategies for recycled plastics to adjust the process from melt pressure. The methodology designed in this work follows four main steps (cf. Figure 1):

1. Data collection from injection molding experiments and testing of the experiment specimens;
2. Model setup, hyperparameter tuning, and training of each of the prediction models;
3. Model validation is conducted by introducing hold-out data and evaluating each model’s ability to predict quality responses;
4. Process optimization to predict the processing parameters associated with each quality response input;
5. All machine learning algorithms were coded using Python version 3.10.12, with Scikit-learn version 1.3.2 used for the implementation of regression models.

### 2.2. Model Training and Validation

For each method, the data were split into 3 categories: training data, testing data, and data for validation. The training and testing data comprise 80% of the samples collected (80 samples, 4 different MFRs) and further split the data into 80% for training (64 samples) and 20% (16 samples) for testing. This 80/20 split follows standard machine learning practices, ensuring sufficient training data (64 samples) for robust learning while retaining an adequately sized test set (16 samples) to evaluate model performance on unseen data. Additionally, a separate validation set of 20 samples was reserved to assess model effectiveness on completely unseen material variations, further ensuring generalization. The last 20% of the data is used to measure the model’s effectiveness and comprises a combination of inputs and responses that the model has not seen before. The data were introduced into the model in two ways. The first uses the MFR values of the material, which were tested for each material lot. The second is the apparent viscosity values calculated using pressure curves and in-mold sensors for each cycle.

The first step (Step 1, Figure 1) was collecting and testing the injection-molded parts. As Step 2 (cf. Figure 1) shows, the collected data were split randomly into an 80/20 split for the model creation and training. Following the training, the models were evaluated by measuring the mean absolute error (MAE), mean squared error (MSE), and R^2^ values using the following equations:

(3) $$MAE=\frac{1}{n}\sum_{i=1}^{n}\left|y_{i}-{\hat{y}}_{i}\right|,$$

(4) $$MSE=\frac{1}{n}\sum_{i=1}^{n}{\left(y_{i}-{\hat{y}}_{i}\right)}^{2}$$

where *y_i_* represents the experimental or observed value and ${\hat{y}}_{i}$ represents the predicted value. MSE was chosen as a primary metric to penalize larger errors, while MAE was used to provide an intuitive measure of average deviation.

The trained machine learning models were then validated (Step 3, Figure 1) by evaluating their effectiveness at predicting part quality parameters (yield stress, modulus, ultimate elongation, and part weight) using hold-out data (material MFR, apparent viscosities, trigger times, and pressures) from a pressure-controlled injection molding process, as seen in step three of the flow chart. This step was crucial to determining the best material viscosity parameter (i.e., MFR or APV). When using MFR, the model did not consider the shot-to-shot variation. In contrast, when using the APV, the model measures the material flow for each cycle. The accuracy of the predicted quality response determined the model’s effectiveness compared to the actual experimental value during the validation step and the minimization of the MAE and MSE values.

Once the model was validated, the optimization component was introduced (Step 4, Figure 1). The objective of the optimization was that of predicting process parameters for a P-Control molding process (pressures, trigger time) when using an rPP of different viscosity while having specific quality parameters as a target (i.e., yield stress, modulus, ultimate elongation, part weight). The upper and lower limits of the variable inputs, P_act_ and t_trig_, were taken as the maximum and minimum values of the 80 samples used to train the model. Additionally, the step size for the P_set_ variable was 5 bar (58 total P_act_ values ranging from 335 bar to 625 bar), and the step size for t_trig_ was 0.1 s (10 total t_trig_ values ranging from 2 s to 3 s) for a total of 638 combinations. Then, for a specific material MFR or APV and a selected quality parameter, the model computes all possibilities and produces a combination of P_act_ and t_trig_, resulting in the lowest error. The predicted values were compared to values obtained experimentally to validate the model’s effectiveness.

### 2.3. Data Collection

The injection molding experiments were performed using two recycled polypropylenes with varying melt flow rates and three different blends of the same grades. The base materials were a non-woven PP (Green Isoplen Y900R, SER North America, Anderson, IN, USA) and a clear recycled BOPP (Green Isoplen C200R, SER North America, Anderson, IN, USA). More information about the materials is provided in previous research from the authors [30]. Table 2 shows the melt flow rate for each material. The injection molding experiments were performed on a fully electric 130-ton machine (Milacron-FANUC Roboshot α-S130iB, Cincinnati, OH, USA). The mold used for the experiments was a two-plate cold runner mold with a 2 mm thick spiral flow cavity. The mold was instrumented with three 2.5 mm direct pressure transducers (Kistler 6182B, Winterthur, Switzerland) flush mounted along the flow path. Additionally, a data acquisition unit integrated within the injection molding machine was used to monitor the sensor signals with a time step of 0.001 s after triggering from the screw forward movement.

The experiments were performed using a pressure-controlled (P-Ctrl) injection molding strategy provided by iMFLUX, Inc. (P&G, Cincinnati, OH, USA) [31,32]. Previous work from the authors has shown that pressure-controlled injection molding is a viable alternative to conventional velocity-controlled injection molding. The authors reported improved tensile parameters, reduced overall molding pressures, and decreased energy consumption [33]. The functionality of the P-Ctrl system and the injection molding experiments used as the base for this work are explained in greater detail in other works by the authors [30].

The combination of experimental runs used for the training and validation of the models can be found in Table 3. The t_trig_ relates to the activation of P-Ctrl’s AV feature, a trigger time of 2 s signifies that the melt must reach the target location in the cavity at 2 s, and therefore the pressure setpoint will be adjusted to reach that location at the given time. That adjusted pressure setpoint is known as P_act_ and is a deviation from the pressure setpoint chosen during the process setup. Table 4 shows the 20 samples used for the validation and optimization problem. These samples were fabricated using the material with an MFR of 14. This material was selected for prediction and optimization as it lay in the middle of the MFR range and would allow the machine learning algorithm to train with data surrounding it. The main difference in these 20 samples is that the first run had a faster melt front velocity due to the shorter trigger time, Trig_2_. In contrast, the second run had a slower melt front velocity due to a longer trigger time, Trig_3_, which resulted in different APV values.

### 2.4. Multivariate Regression Model

Two multivariate regression models were created using the Scikit learn library in Python [34]. The first model is a linear regression model and the second one is a polynomial regression model, ranging from second to fourth degrees. For both models, the data were split as stated in step 2. For the polynomial regression model, Grid Search with 5-fold cross-validation was used to determine the optimal degree. A pipeline of polynomial feature transformation followed by linear regression was implemented, and a linear regression model was trained for each degree. The mean validation score was calculated using the R^2^ score, and the degree with the highest validation score was selected to balance model complexity and prediction accuracy. Following this, the coefficients for each independent variable were obtained and the model performance was assessed using R^2^, MSE, and MAE. The remaining 20 samples were kept separately, were arithmetically averaged based on their inputs, and were implemented similarly to the ANN for both approaches.

### 2.5. Artificial Neural Networks

In this work, the ANNs were implemented using TensorFlow version 2.15.0 and Keras version 2.15.0. The neural network architecture comprised 6 hidden layers with 32 neurons per layer. The ANN architecture was determined through empirical tuning to balance model complexity and predictive performance. A six-layer structure with 32 neurons per layer was chosen as it provided the best trade-off between accuracy and computational efficiency. Testing alternative architectures showed that fewer layers led to underfitting, while deeper networks increased the training time without meaningful performance improvements. The final architecture was selected based on validation performance, and early stopping was implemented to prevent overfitting. This configuration is consistent with prior work in ANN-based process modeling for materials science and injection molding. Additionally, a Rectified Linear Unit (ReLU) activation function was used with a ‘he_normal’ initializer and an Adaptive Moment Estimation (Adam) Optimizer with a 0.001 learning rate. The ‘he_normal’ initializer was specifically chosen because it draws weights from a normal distribution scaled by the number of input neurons. This helps maintain stable activation magnitudes throughout the network, reducing the risk of vanishing or exploding gradients. As a widely recommended method for deep networks using ReLU, it enhances convergence speed and overall training stability. Lastly, early stopping was implemented to prevent the overfitting of the models, with a patience parameter of 20, meaning training continued for up to 20 epochs without improvement before stopping. A minimum improvement threshold (min_delta) of 0.00001 was set to ensure only meaningful reductions in validation loss were considered. The mode was set to ‘auto’ to determine whether to minimize or maximize the tracked metric. Model performance was assessed on the MAE and MSE during training and validation. Specifically, the difference between the training and validation MAE and MSE was evaluated. Appendix A shows a sample script used to create and validate the ANN model.

After training the model, the data for the rPP with hol MFR, t_trig_, P_act_, and APV were introduced, and the hold-out quality parameter was predicted and compared to experimental values (step three). The % error, MAE, and MSE were once again evaluated to determine the model’s ability to predict hold-out data.

Following the model’s validation, the potential combinations of variable inputs and quality responses were introduced. Following this, the model calculated the error each combination would provide for each constant input, and the lowest resulting error was deemed the predicted output (step four). To evaluate the models, the %error for each predicted combination was measured.

## 3. Results and Discussion

### 3.1. Experimental Results

Table 5 shows a summary of the training data used for each model, and all of the raw data can be found in Appendix B. The table shows that a wide range of data was used for the training of the models, particularly for the yield stress and modulus. Additionally, the modulus had the overall highest variability within each of the runs and the APV value had a significant variation for a run with a material MFR of 8 and a 3 s trigger time. Indeed, the adaptive process control technology was unable to stabilize during this run, which caused significant variation within it. The reason is that a longer trigger time slows the melt front velocity down significantly, which can result in the approaching the melt front velocity approaching the Newtonian region, which makes it more susceptible to changes in shear rate, which increases shot-to-shot variation [15]. Additionally, the material with an MFR of 8 was a blend with increased content of the low MFR material added additional variation to the mix.

For all of the models evaluated, the experimental values for the predicted quality parameters can be found in Table 6. As seen in this table, the modulus showed the highest variation during the experimental procedure, whereas the weight and yield stress were relatively consistent. Additionally, it can be seen that the Trig_3_ runs showed greater variation than Trig_2_, similar to the results reported in Table 5.

### 3.2. Multivariate Regression Model Creation and Evaluation

Determining the ideal degree for the polynomial regression model was performed by assessing the mean score of each degree achieved during cross-validation. This step was crucial as selecting a degree that is too low will result in significant interactions being skipped, whereas selecting a degree that is too high can result in model overfitting and poor performance. Table 7 shows the coefficient of determination for each of the models with the different inputs. From the R^2^ values, the yield stress and modulus performed well for all models, with the polynomial regression model having a slightly higher R^2^. Ultimate elongation performed the worst for the linear regression with slightly higher performance for the polynomial regression model as the additional interactions were able to be captured by the higher order degree from the polynomial model.

Comparing the MFR model to the APV model, one can see an increase in R^2^ for the viscosity model when looking at yield stress and modulus but a decrease in part weight R^2^ for the linear regression model. The reason is that when using the MFR to train the model, there is less variation within the data itself, as using the MFR essentially reduces the training data to 8 runs with 10 identical inputs Teach. This allows responses that do not have large variance across runs (such as part weight fluctuated between 13.43 g and 13.17 g across runs (c.f. Appendix B for all raw data) to have their inputs predicted as there are fewer combinations to select. On the other hand, the APV model has 80 different input combinations used during training, which, when the responses are similar, could result in uncertainty when trying to make predictions but also increase the amount of noise and outliers, which could affect the model performance.

### 3.3. Artificial Neural Network Setup and Evaluation

**Figure 2:**
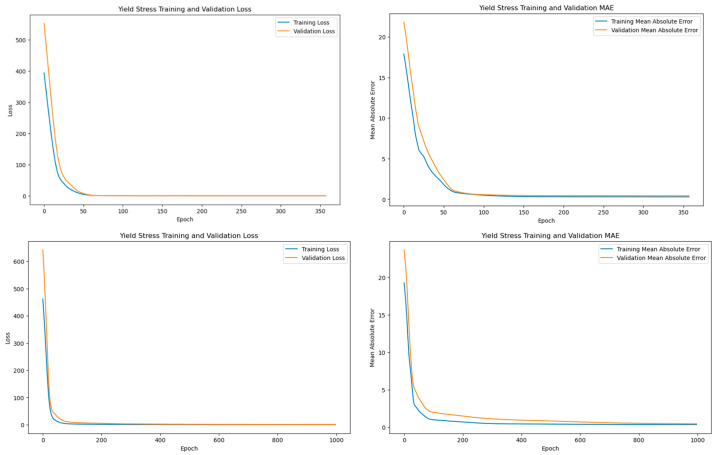
Training and validation plots for the yield stress response variable for ANN using MFR (**top row**) and ANN using APV (**bottom row**).

A neural network model was created for each of the quality response variables; however, for simplicity, only plots for the yield stress response will be shown. Figure 2 show the training and validation loss and the training and validation mean absolute error for the yield stress response variable for the Trig_2_ runs using MFR (Figure 2 top row) and APV (Figure 2 bottom row). These plots highlight the importance of hyperparameter tuning and data splitting as a model that is over- or under-fit. Ideally, as the model continues to learn, the loss and the mean absolute error (MAE) decrease as the number of epochs increases while maintaining a minimum difference between the training and validation errors. A large gap between the training and validation curves could potentially signify poor model performance in the form of over- or underfitting. Additionally, as seen in the figure, both the MFR and APV models show no signs of overfitting thanks to the early stopping implemented (MFR stopped at 376 epochs, while APV stopped at 998 epochs). The significant difference in epochs reflects the complexity of the datasets, the APV model required more training cycles to capture the greater variability in viscosity-related parameters, whereas the MFR model, with more consistent data, converged more quickly. This extended training allowed the APV model to better generalize to the increased shot-to-shot variation present in the dataset. The difference in epochs means that the model with the APV underwent the training process 622 more times than the model run with the MFR data. The additional epochs allow the model to learn from the data more extensively and give the model a chance for better convergence and, thus, a better understanding of the data’s patterns. These additional epochs could have been necessary for the model to better understand the data due to the APV creating that additional variation within the dataset.

Table 8 shows the final values for the loss and MAE for each response for both the MFR and APV models. As can be seen, the magnitude of the losses varies significantly for each response variable, and this magnitude shows the average discrepancy between the predicted response and the actual response.

Comparing the model run using MFR and the model using APV, it can be observed that APV model performed better for the modulus and the yield stress response. However, the MFR model performed better for the ultimate elongation and part weight model. Indeed, when using the MFR to train the model, there is less variation within the data itself, as using the MFR essentially reduces the training data to 8 runs with 10 identical inputs each. This followed the same trend and reasoning as the regression models in which introducing additional variation to the dataset increased the noise.

### 3.4. Validation of the Models

#### 3.4.1. Multivariate Regression Models

After evaluating the models and their coefficients, the hold-out data used for the ANN were introduced similarly for each regression model. The % error for each of the predicted quality responses and the experimental values for each regression model can be seen in Table 9. From the table, the APV model performs significantly better for the yield stress, modulus, and ultimate elongation response. A best-performing model could not be identified for the part weight response. Comparing the regression models, the linear model performs equally or better than the polynomial model. Whereas, the MFR model outperformed the linear model for the yield stress and ultimate elongation. The polynomial model notably performs very poorly at predicting the modulus when using the MFR data with errors greater than 30%. The error decreased to under 9% when using the APV data. The reason for this is that the polynomial models are known for performing quite well at predicting data that is close to the training data; however, the models tend to collapse when using data outside of the training set. When using MFR, the training data becomes limited, and therefore there are fewer unique training combinations, thus limiting the model. When using the APV, the number of unique training combinations increases, thus allowing the model to be better prepared for more variable validation data.

#### 3.4.2. Artificial Neural Network

Once the models were trained, the hold-out data were introduced (inputs c.f. Table 4). Following this, the model was re-run to predict the values of the hold-out data. Table 10 shows the average % error between the predicted quality response and the actual response seen in Table 6. As seen in the table, the Trig_2_ runs all fall within 10% error, whereas the Trig_3_ runs tend to show increased error up to 15%. This was expected due to the increased variation in experimental data (larger standard deviations in Table 6). Comparing the MFR model to the APV model, one can see that for yield stress, ultimate elongation, and modulus, there is no clear trend as to which model is most beneficial. However, the MFR model significantly reduces the % error for part weight, which was expected due to the better training results seen in Table 7. From the training results, the expected result would be a minimal increase in validation accuracy for the APV model. However, this was observed in just a few instances, likely due to the increase in training performance being insufficient to warrant a significant decrease in error percentage. Indeed, the amount of variation within each of the runs caused the model to fail to recognize certain trends in the data when training, and the creation of different combinations created additional noise.

#### 3.4.3. Comparison Between Models

**Figure 3:**
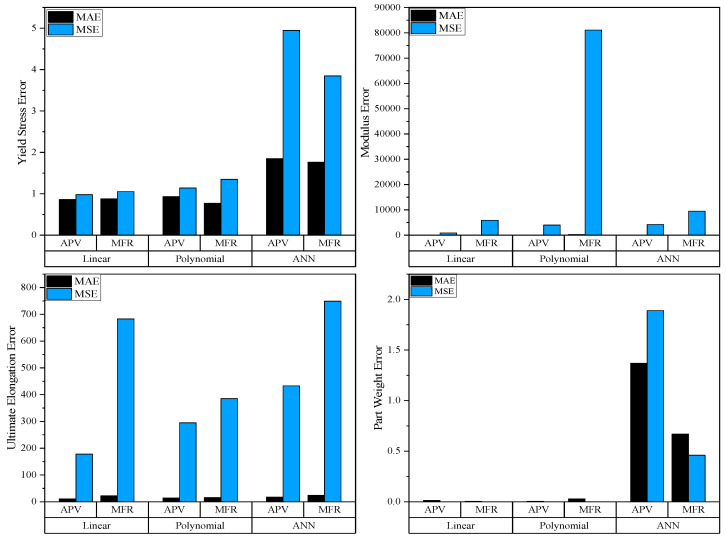
Errors between experimental and predicted responses for all models.

The comparison between the regression models and the ANN can be seen in Figure 3, which compares the MAE and MSE between the three models and the different datasets. As seen in the figure, the MSE is significantly larger for the yield stress, modulus, and ultimate elongation, which signifies that the predictions are distributed relatively evenly amongst the true values. For the weight, however, it can be seen that the MAE is larger than the MSE for all but the ANN using MFR data. This signifies that there were a few outliers in the data that heavily influenced the model. Additionally, it can be seen that the ANN performed worse for all responses except for Modulus (Polynomial with MFR data was the worst performer). Comparing these results to the literature, it can be seen that similar results on the performance between ANN and polynomial regression were found, and the authors concluded that there was no best model for each case [22]. Additionally, they determined that hyperparameter tuning and the data selected for the training of the models were key to preventing overfitting and the best prediction behaviors. However, other authors have compared ANNs to regression analysis and their results show a clear advantage to using an ANN for predicting relationships between inputs and outputs using similar size datasets [35]. The main difference is the amount of variation within the dataset as the authors only used a single virgin polypropylene, and their response variable was dimension stability. It is hypothesized that the complex relationships between the quality responses and the processing inputs were not adequately captured, and additional data would be required to fully grasp the relationship and make more accurate predictions. However, except for part weight, the ANN performed quite similarly to the regression models, with errors below 6% for the Trig_2_ runs.

The different datasets also did not seem to provide a clear trend for the ANN, as using the APV data proved advantageous for the modulus and ultimate elongation models, and the MFR data proved more accurate for the part weight and yield stress models. For the linear and polynomial regression models, the APV showed vast improvements, particularly for the polynomial model being able to reduce the MSE of the modulus model by 99.65% (from 81,000 to 280).

### 3.5. Process Input Optimization

Following the validation of each machine learning model, the hold-out data were reorganized for an optimization algorithm so that the quality responses would become inputs and the processing variables would be the resulting outputs. Table 11 shows the data breakdown for the machine learning optimization. Each response model created in Section 3.1 was used to predict the Ttrig and the Pact used for the hold-out material with a given MFR and a calculated APV. The ideal combination of T_trig_ and the P_act_ was conducted by evaluating the error of each of the 638 combinations, as elaborated in Section 2.1. Table 12 shows the average combination and % error of T_trig_ and P_act_ determined by each model for each response.

From Table 12, all models show at least a 12% error in predicting the trigger time that was used to conduct the experiments. However, the models are more accurate at predicting the pressures that were seen during experiments. A conclusion cannot be drawn on which model performs the best at predicting the overall mechanical properties and part weights, as each model struggles to find the accurate processing input that was used, with errors ranging from 3% to 25% for all models. The error in the optimization of the process inputs is much larger than that seen during the validation of the models, which shows that although the models showed adequate training performance, perhaps additional feature engineering is necessary to fully capture the relationship between the inputs and the outputs. Indeed, the relationship between plastics properties and processing inputs is complex and therefore additional input data may be necessary for the model to fully understand [15].

**Figure 4:**
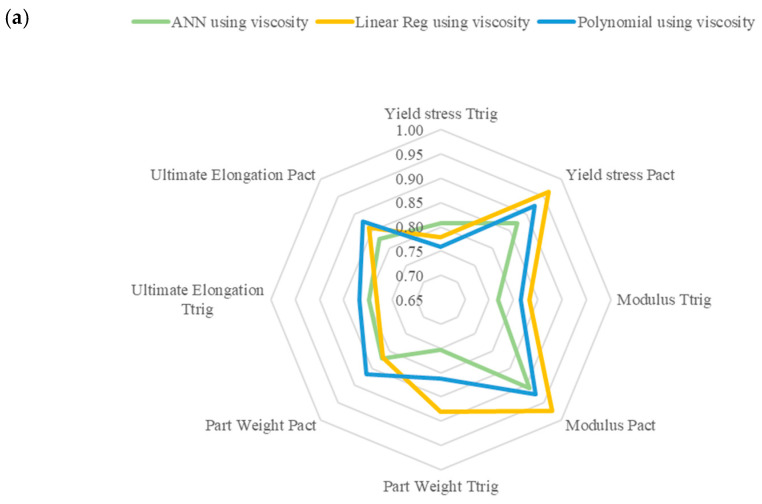

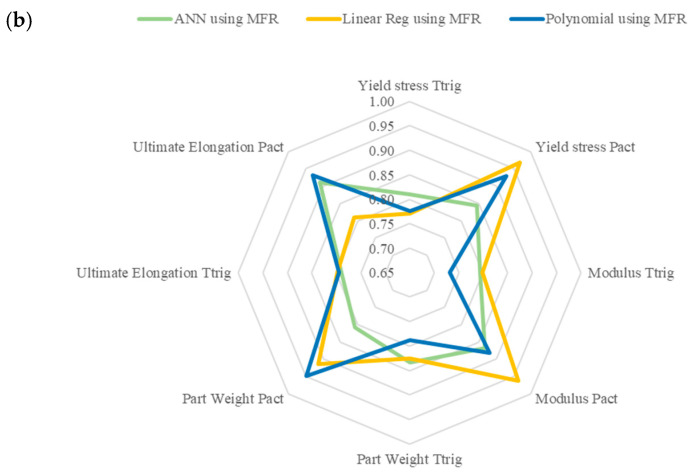
Radar plot showing the performance of each model for each predicted input variable using (**a**) APV and (**b**) MFR data.

Additionally, the dataset used had no real impact on the optimization problem, as both datasets appear to perform similarly for all models. This could also signify the need for feature reassessment as the difference between the two datasets was evident during the model training and validation. Figure 4a shows a radar chart highlighting the combination of inputs that give the best performance for the APV dataset and Figure 4b for the MFR dataset. The closer to the outside of the radar, the more accurate that specific model is at predicting the response since the data plotted is one error from Table 12. As seen in the figures, the model using MFR data can more accurately predict the Pact value; however, in doing so, it is unable to predict the $T_{trig}$. The model using the APV data is more accurately able to predict the $T_{trig}$ value; however, in doing so, it sacrifices accuracy in $P_{act}$.

### 3.6. Validation of Input Prediction

As Section 3.5 focuses on the prediction of processing inputs based on specific mechanical properties as responses, this section focuses on determining whether the predicted inputs can indeed meet the required mechanical properties. Therefore, four additional runs were performed with the processing inputs predicted in Section 3.5 focusing on the two different datasets and with yield stress as the target mechanical property, Table 13. highlights the setup for the 4 validation runs.

The samples were fabricated following the same procedure presented in Section 2.3. After the samples were fabricated, tensile testing was performed following ASTM D638 [36], which tested five samples for each run. The yield stress values obtained using the model-suggested inputs were then compared to the original yield stress values, as seen in Table 14. The results show that using the model inputs resulted in yield stresses lower than the original yield stress values. However, the error was within ~6% across each of the datasets, which showed the resilience of the material to obtain the same mechanical properties using different processing inputs. Moreover, it showed the capabilities of machine learning to predict inputs that provide accurate responses.

## 4. Conclusions

This work investigated the capabilities of an ANN, linear regression, and polynomial regression model to predict quality responses based on processing inputs in injection molding. The following are the main takeaways from the work:

- Processing inputs were predicted based on material properties and quality responses for a P-Ctrl injection molding process using 5 blends of recycled polypropylene;
- The work focused on the training, validation, and optimization of these models and the ability of each of the models to predict different outputs based on complex material and processing relationships;
- The research explored tuning of the models is important to optimize the prediction of the behavior of challenging recycled materials. The proposed strategy could lead to an increase in their usage across different industries;
- Models created could accurately predict the yield stress, ultimate elongation, and part weight to within 5% error for the linear and polynomial models and 10% error for the ANN with the Trig_2_ run;
- The predictions for the modulus were far less accurate with a % error of ~11% for the ANN and the linear regression models and of ~40% for the polynomial model. The modulus, as shown in the data, is a property that tends to show high variation across and within each of the runs. Therefore, the models struggle to find the proper trends, leading to larger errors in the predicted data;
- The differences between models’ performances can be attributed to data variability across different features. Future work will focus on expanding the analysis for different materials to allow more in-depth analysis of the sources of error;
- The optimization problem results showed no single methodology to be superior for all responses, as specific models performed better for particular ones. However, the overall results of predicting the processing inputs, T_trig_ and P_act_, were much worse, with errors between 3–25% depending on the response and the model;
- Future work will focus on adding a feature reassessment to identify other combinations of inputs that could better capture the complex relationships seen during the process. Additionally, alternative machine learning methodologies will be evaluated, including ensemble methods, such as Random Forest and Gradient Boosting, which could improve generalization and better capture the complex relationships in the data.

## Figures and Tables

**Table 1:** Previous research work reported on the use of machine learning in injection molding.

Reference	Machine Learning Technique	Input Variables	Quality Metric
Heinisch et al. [22]	ANN	Simulation based, injection time, cooling time, packing pressure, packing time, melt and mold temperature	Length, width, weight
Yin et al. [16]	BP neural network	Mold and melt temperature, packing pressure, packing time, cooling time	Warpage
Lee et al. [17]	ANN, transfer learning	Combination of experimental and simulation based, geometric features and processing features	Weight
Xu et al. [18]	ANN combined with particle swarm optimization	Simulation based, mold and melt temperature, injection velocity, compression distance, force, velocity and waiting time	Impact performance (von mises stresses)
Yousef et al. [19]	ANN	Strain, blending ratio	Tensile performance and curves
Ozcelik, et al. [20]	ANN, ANOVA, genetic algorithm	Simulation based, mold and melt temperature, packing pressure, packing time, gate type, and gate location	Warpage
Kenig et al. [21]	ANN, multivariate regression	Melt and mold temperature, packing pressure, injection time, cooling time	Tensile modulus
Youssef, et al. [11]	Polynomial regression	Mechanical properties	Prediction accuracy, cost

**Table 2:** Selected material blends and their melt flow rates.

Material	Non-Woven (%)	BOPP (%)	MFR (g/10 min)
Material A	80	20	32
Material B	50	50	14
Material C	20	80	8
Material D	100	0	50
Material E	0	100	5

**Table 3:** Complete list of runs for training models.

Run	Melt Flow Rate (g/10 min)	T_trig_ (s)	P_act_ (bar)
1	32	2	415
2	32	3	382
3	8	2	563
4	8	3	538
5	50	2	367
6	50	3	336
7	5	2	624
8	5	3	598

**Table 4:** DoE runs used for validation.

Run	MFR (g/10 min)/APV (Pa-s)	T_trig_ (s)	P_act_ (bar)
Trig_2_	14/184	2	483
	14/182	2	
	14/183	2	
	14/190	2	
	14/186	2	
	14/181	2	
	14/177	2	
	14/177	2	
	14/186	2	
	14/194	2	
Trig_3_	14/319	3	457
	14/335	3	
	14/314	3	
	14/338	3	
	14/304	3	
	14/407	3	
	14/378	3	
	14/325	3	
	14/320	3	
	14/348	3	

**Table 5:** Summary of data used to train the models.

Material MFR (g/10 min)	Trigger Time (s)	APV (Pa-s)	Yield Stress (MPa)	Modulus (MPa)	Ultimate Elongation (%)	Part Weight (g)
5	2	277 ± 1.2	32.57 ± 0.12	1326 ± 11	340 ± 7.5	13.21 ± 0.003
5	3	497 ± 4.4	32.81 ± 0.38	1353 ± 14	327 ± 8	13.19 ± 0.002
8	2	233 ± 3.5	29.2 ± 0.45	1198 ± 35	319 ± 9	13.21 ± 0.002
8	3	680 ± 270	28.8 ± 0.77	1163 ± 59	319 ± 13	13.18 ± 0.003
32	2	135 ± 2.6	12.7 ± 0.22	333 ± 12	395 ± 5	13.21 ± 0.003
32	3	263 ± 10	13.2 ± 0.18	357 ± 11	397 ± 6	13.18 ± 0.002
50	2	102 ± 1.1	11.62 ± 0.1	287 ± 6	347 ± 24	13.19 ± 0.002
50	3	181 ± 1.5	11.92 ± 0.09	302 ± 14	370 ± 10	13.17 ± 0.003

**Table 6:** Responses used as the constant input for the optimization of each model (MFR of 14).

Material MFR (g/10 min)	Trigger Time (s)	APV (Pa-s)	Yield Stress (MPa)	Modulus (MPa)	Ultimate Elongation (%)	Part Weight (g)
5	2	277 ± 1.2	32.57 ± 0.12	1326 ± 11	340 ± 7.5	13.21 ± 0.003
5	3	497 ± 4.4	32.81 ± 0.38	1353 ± 14	327 ± 8	13.19 ± 0.002
8	2	233 ± 3.5	29.2 ± 0.45	1198 ± 35	319 ± 9	13.21 ± 0.002
8	3	680 ± 270	28.8 ± 0.77	1163 ± 59	319 ± 13	13.18 ± 0.003
32	2	135 ± 2.6	12.7 ± 0.22	333 ± 12	395 ± 5	13.21 ± 0.003
32	3	263 ± 10	13.2 ± 0.18	357 ± 11	397 ± 6	13.18 ± 0.002
50	2	102 ± 1.1	11.62 ± 0.1	287 ± 6	347 ± 24	13.19 ± 0.002
50	3	181 ± 1.5	11.92 ± 0.09	302 ± 13	370 ± 10	13.17 ± 0.003

**Table 7:** R^2^ values for each of the multivariate regression models.

Response	MFR Model		APV Model	
	R^2^	R^2^/Degree	R^2^	R^2^/Degree
	Linear Regression	Polynomial Regression	Linear Regression	Polynomial Regression
Yield Stress	95.6	98.2/4	96.5	98.9/3
Modulus	93.5	96.5/4	94.8	98.9/3
Ultimate Elongation	12.3	55.3/3	44.8	69.6/3
Part Weight	58.1	54.9/2	56.7	58.2/2

**Table 8:** Final validation and training losses and MAE for each response variable.

Response	Training/Validation	MFR Model		APV Model	
		Final MAE	Final Loss	Final MAE	Final Loss
Yield Stress	Training	0.432	0.387	0.348	0.208
	Validation	0.756	1.34	0.451	0.523
Modulus	Training	31.1	2187	22.46	1016
	Validation	54.55	6609	38.68	2824
Ultimate Elongation	Training	15.01	560	19.48	915
	Validation	17.03	448	21.7	637
Part Weight	Training	0.0043	0.00003	0.027	0.0016
	Validation	0.0057	0.00005	0.128	0.06

**Table 9:** % error between predicted quality response and experimental values for each regression model.

		Linear Model		Polynomial Model	
Run	Response	Model	% Error	Model	% Error
Trig_2_	Yield Stress	APV	2.38	APV	2.48
		MFR	5.85	MFR	2.09
	Modulus	APV	1.06	APV	5.28
		MFR	11.59	MFR	31.8
	Ultimate Elongation	APV	1.43	APV	3.76
		MFR	5.81	MFR	2.45
	Part Weight	APV	0.07	APV	0.01
		MFR	0.02	MFR	0.22
**Run**	**Response**	**Model**	**% Error**	**Model**	**% Error**
Trig_3_	Yield Stress	APV	2.75	APV	1.56
		MFR	5.54	MFR	2.52
	Modulus	APV	0.68	APV	8.5
		MFR	11.34	MFR	44.09
	Ultimate Elongation	APV	1.28	APV	2.54
		MFR	5.9	MFR	5.32
	Part Weight	APV	0.08	APV	0.02
		MFR	0.01	MFR	0.22

**Table 10:** % error between predicted quality response and experimental values for both ANN models.

Run	Response	Model	% Error
Trig_2_	Yield Stress	APV	3.37
		MFR	5.02
	Modulus	APV	10.78
		MFR	5.02
	Ultimate Elongation	APV	5.17
		MFR	5.74
	Part Weight	APV	9.6
		MFR	5.72
**Run**	**Response**	**Model**	**% Error**
Trig_3_	Yield Stress	APV	14.99
		MFR	12.6
	Modulus	APV	3.87
		MFR	10.6
	Ultimate Elongation	APV	3.85
		MFR	7.61
	Part Weight	APV	11.15
		MFR	4.23

**Table 11:** Model inputs for the optimization problem.

Ultimate Elongation (%)	Yield Stress (MPa)	Modulus (MPa)	Part Weight (g)	MFR (g/10 min)/Viscosity (Pa-s)
356	20.59	762	13.21	14/184
345	20.12	750	13.21	14/182
368	20.04	747	13.22	14/183
345	20.53	770	13.22	14/190
349	21.10	776	13.22	14/186
344	20.64	776	13.21	14/181
365	19.93	737	13.21	14/177
369	19.56	722	13.22	14/177
355	20.04	730	13.21	14/186
379	19.60	729	13.21	14/194
359	20.26	755	13.20	14/319
369	19.88	743	13.20	14/335
347	20.21	758	13.20	14/314
330	19.90	730	13.19	14/338
365	19.82	725	13.20	14/304
372	20.63	762	13.20	14/407
361	19.33	684	13.19	14/378
343	19.53	711	13.20	14/325
374	20.19	744	13.20	14/320
372	19.25	710	13.19	14/348

**Table 12:** Predicted input parameters for each model.

ANN Model								
Data Used	Yield Stress		Modulus		Ultimate Elongation		Part Weight	
	T_trig_ %error	P_act_ %error	T_trig_ %error	P_act_ %error	T_trig_ %error	P_act_ %error	T_trig_ %error	P_act_ %error
APV	19.20%	12.80%	23.30%	9.20%	20.20%	17.20%	24.70%	17.80%
MFR	19%	15.60%	20.80%	13.30%	21.10%	9.0%	16.70%	19.20%
**Linear Model**								
Data Used	Yield Stress		Modulus		Ultimate Elongation		Part Weight	
	T_trig_ %error	P_act_ %error	T_trig_ %error	P_act_ %error	T_trig_ %error	P_act_ %error	T_trig_ %error	P_act_ %error
APV	22.10%	3.60%	16.80%	2.60%	21.80%	14.00%	12.00%	18.24%
MFR	23%	3.20%	20.10%	3.70%	17.40%	8.7%	20.30%	19.00%
**Polynomial Model**								
Data Used	Yield Stress		Modulus		Ultimate Elongation		Part Weight	
	T_trig_ %error	P_act_ %error	T_trig_ %error	P_act_ %error	T_trig_ %error	P_act_ %error	T_trig_ %error	P_act_ %error
APV	24.20%	7.70%	18.60%	7.40%	18.30%	12.30%	19.70%	13.40%
MFR	22.40%	7.00%	26.80%	11.90%	20.60%	7%	21.20%	5.20%

**Table 13:** Setup of validation runs.

	Original		Predicted	
Dataset	Trigger	Pressure	Trigger	Pressure
Viscosity	2	483	2.5	512
Viscosity	3	457	2.6	522
MFR	2	483	2.46	455
MFR	3	457	2.55	468

**Table 14:** Yield stress comparison between original inputs and the model predicted inputs.

	Yield Stress (MPa)	
Dataset	Model Inputs	Actual Inputs
Viscosity	19.06	20.22
Viscosity	18.79	19.9
MFR	19.02	20.22
MFR	19.79	19.9

## Data Availability

The raw data supporting the conclusions of this article will be made available by the authors upon request.

## Appendix B. Raw Data

**Table A1:** Raw data used for model training, validation, and optimization.

Material MFR (g/10 min)	Trigger Time (s)	Actual Pressure (bar)	Ultimate Elongation (%)	Yield Stress (MPa)	Modulus (MPa)	Part Weight (g)	APV (Pa-s)
5	2	624	334.36	32.64	1361.83	13.21	275.48
5	2	624	353.54	32.66	1353.72	13.22	276.97
5	2	624	335.73	32.23	1323.97	13.21	277.46
5	2	624	326.65	32.51	1320.19	13.21	275.17
5	2	624	347.85	32.66	1332.20	13.20	277.23
5	2	624	321.91	32.77	1320.84	13.21	274.91
5	2	624	339.89	32.90	1334.81	13.22	274.12
5	2	624	335.37	32.52	1318.86	13.21	277.72
5	2	624	335.93	32.75	1319.47	13.21	282.95
5	2	624	368.35	32.10	1275.79	13.22	276.37
5	3	598	332.31	33.07	1342.81	13.19	509.84
5	3	598	335.70	33.40	1341.64	13.19	494.71
5	3	598	349.49	31.15	1303.58	13.19	473.78
5	3	598	306.55	34.08	1402.71	13.19	500.30
5	3	598	331.19	32.60	1330.13	13.18	496.31
5	3	598	337.84	31.89	1338.42	13.19	497.07
5	3	598	324.93	32.91	1379.63	13.18	500.34
5	3	598	320.70	33.14	1349.97	13.18	501.40
5	3	598	297.53	33.08	1378.71	13.19	498.53
5	3	598	336.11	32.80	1367.48	13.18	493.08
8	2	563	347.15	28.42	1128.57	13.22	229.50
8	2	563	318.19	28.55	1147.12	13.21	232.38
8	2	563	327.12	28.51	1115.59	13.21	223.12
8	2	563	334.80	28.54	1155.11	13.21	226.64
8	2	563	341.88	27.72	1121.72	13.22	244.75
8	2	563	306.98	30.17	1284.43	13.21	241.79
8	2	563	315.01	29.80	1245.28	13.21	232.20
8	2	563	296.44	29.63	1217.40	13.21	227.24
8	2	563	291.60	30.72	1303.36	13.21	243.21
8	2	563	310.26	29.94	1264.10	13.21	231.96
8	3	538	304.85	30.10	1284.07	13.19	419.12
8	3	538	289.77	30.85	1280.18	13.19	442.14
8	3	538	320.12	29.82	1246.01	13.19	447.77
8	3	538	286.17	30.36	1303.06	13.19	402.20
8	3	538	278.10	30.47	1289.62	13.20	441.22
8	3	538	341.33	27.45	1047.07	13.18	969.45
8	3	538	339.06	28.16	1076.78	13.19	974.48
8	3	538	333.76	27.29	1049.93	13.19	959.64
8	3	538	343.27	26.86	1035.26	13.18	1091.14
8	3	538	351.63	26.96	1022.20	13.18	653.57
14	2	483	356.87	20.59	762.45	13.21	184.68
14	2	483	345.65	20.12	750.41	13.21	182.21
14	2	483	368.24	20.04	747.24	13.22	183.65
14	2	483	345.34	20.53	770.93	13.22	190.12
14	2	483	349.34	21.10	776.26	13.22	186.66
14	2	483	344.47	20.64	776.86	13.21	181.36
14	2	483	365.18	19.93	737.49	13.21	177.64
14	2	483	369.39	19.56	722.31	13.22	177.65
14	2	483	355.92	20.04	730.33	13.21	186.52
14	2	483	379.80	19.60	729.58	13.21	194.29
14	3	457	359.06	20.26	755.07	13.20	319.25
14	3	457	369.96	19.88	743.02	13.20	335.31
14	3	457	347.07	20.21	758.87	13.20	314.59
14	3	457	330.97	19.90	730.88	13.19	338.14
14	3	457	365.56	19.82	725.93	13.20	304.05
14	3	457	372.34	20.63	762.41	13.20	407.50
14	3	457	361.73	19.33	684.55	13.19	378.87
14	3	457	343.82	19.53	711.29	13.20	325.27
14	3	457	374.85	20.19	744.37	13.20	320.52
14	3	457	372.21	19.25	710.97	13.19	348.57
32	2	415	397.97	13.58	374.46	13.21	135.84
32	2	415	398.13	12.91	337.80	13.21	132.26
32	2	415	395.81	11.92	288.90	13.21	130.55
32	2	415	375.94	12.34	300.99	13.20	139.71
32	2	415	395.87	13.05	331.82	13.20	144.78
32	2	415	385.01	12.77	344.96	13.20	138.48
32	2	415	408.92	12.77	336.38	13.21	130.93
32	2	415	410.16	12.74	333.87	13.20	139.93
32	2	415	386.21	12.99	349.26	13.21	130.07
32	2	415	397.15	12.44	327.58	13.20	127.63
32	3	383	380.97	13.87	398.49	13.18	238.31
32	3	383	387.53	13.36	370.07	13.18	261.67
32	3	383	385.43	13.19	362.70	13.18	230.65
32	3	383	418.18	13.03	340.46	13.18	245.37
32	3	383	395.07	13.53	380.87	13.18	271.56
32	3	383	392.74	13.25	365.19	13.18	265.16
32	3	383	411.57	12.63	322.07	13.18	261.61
32	3	383	402.72	12.99	348.56	13.18	269.48
32	3	383	406.46	13.07	350.22	13.19	299.13
32	3	383	386.96	12.69	330.30	13.18	288.72
50	2	367	209.39	11.45	289.17	13.18	103.64
50	2	367	354.55	11.79	297.29	13.19	100.79
50	2	367	353.27	11.69	290.74	13.19	105.12
50	2	367	372.53	11.23	260.94	13.19	101.98
50	2	367	369.58	11.40	276.97	13.19	106.23
50	2	367	372.72	11.91	305.30	13.19	100.50
50	2	367	371.66	11.87	300.16	13.19	99.93
50	2	367	354.12	11.63	279.09	13.18	100.76
50	2	367	338.49	11.60	282.68	13.19	104.05
50	2	367	376.09	11.70	284.47	13.18	100.14
50	3	336	348.24	11.80	299.26	13.18	175.92
50	3	336	362.97	11.58	288.62	13.17	174.99
50	3	336	388.54	12.19	319.45	13.16	180.95
50	3	336	380.54	11.92	303.80	13.17	179.36
50	3	336	390.18	11.72	289.86	13.17	179.80
50	3	336	385.02	11.92	302.17	13.17	180.70
50	3	336	357.50	12.16	316.57	13.16	181.50
50	3	336	330.23	11.94	296.02	13.17	185.16
50	3	336	377.34	11.84	288.48	13.16	185.46
50	3	336	382.42	12.13	312.36	13.17	181.63
